# Design, Development, and Performance Evaluation of Solar Heating System for Disinfection of Domestic Roof-Harvested Rainwater

**DOI:** 10.1155/2015/529527

**Published:** 2015-01-12

**Authors:** O. A. Akintola, A. Y. Sangodoyin

**Affiliations:** ^1^National Horticultural Research Institute of Nigeria, Secretariat Post Office, P.O. Box 29662, Idi-Ishin, Ibadan, Nigeria; ^2^Department of Agricultural and Environmental Engineering, Faculty of Technology, University of Ibadan, Ibadan, Nigeria

## Abstract

A box-type solar heater was designed, constructed, and used to determine the effect of solar heating on quality of domestic roof-harvested rainwater (DRHRW). During testing, naturally contaminated DRHRW was harvested in Ibadan, Nigeria, and released into the system at 93.96 Lh^−1^ (2.61 × 10^−5^ m^3^ s^−1^) in a continuous flow process. Water temperatures at inlet, within the heating chamber, and at outlet from the heating chamber and solar radiation were monitored at 10 min interval. Samples were collected at both inlet to and outlet from the heating chamber at 10 min interval for microbiological analysis. The highest plate stagnation temperature, under no-load condition, was 100°C. The solar water heater attained a maximum operational temperature of 75°C with 89.6 and 94.4% reduction in total viable count and total coliform count, respectively, while *Escherichia coli* and *Staphylococcus aureus* were completely eradicated at this temperature. The solar heater developed proved to be effective in enhancing potability of DRHRW in Ibadan, Nigeria. This may be an appropriate household water treatment technology for developing countries, hence, a way of resolving problem of low quality water for potable uses.

## 1. Introduction

Good quality water is gradually getting beyond the reach of average households in developing countries [1]. This is attributed to low income earnings vis-à-vis the ever increasing cost of other sources of energy for water disinfectant such as kerosene and firewood. The use of chloride solution concentrate has been reported to be an effective means of cleaning vessels for water disinfection and prevention of waterborne diseases [2, 3]. It however does not prevent recontamination. It was therefore suggested that efforts should be made to protect water after treatment up until the point of use [4]. To achieve this, WHO [5] suggested the use of residual chlorine of between 0.2 and 0.5 mg/L. The option is viable in places where public tap supply is in operation. This is far from reality in developing nations due to the collapse of public water supply systems.

Most rural and semiurban settlements do not have the “privilege” of being connected to national electricity grid either. Where they are connected, the erratic supply makes it imperative for cheaper and more reliable source of energy that could be used especially for water pasteurization to be sourced. Solar energy is a free, inexhaustible, and environment-friendly resource [6, 7]. It is estimated that one billion people worldwide do not have access to treated drinking water [8]. Harvesting of solar energy and its use may be a way out of this crisis, not only because of its availability in Nigeria, but mainly due to its relative safety as well, compared to the use of fossil fuels [9].

Solar water heater can be used to pasteurize water thereby destroying harmful food-water microbes including bacteria and viruses when heated to temperatures of about 65°C [10, 11]. Countries that could be considered as having potential for solar cooking must satisfy certain criteria, some of which include high insolation, fuel wood shortages, low per capital income, and high population density. Similarly, the estimated number of potential beneficiaries must be high. These conditions are satisfied by Nigeria with a ranking of five (5) in the list of countries with the highest potential benefit from solar cooking [12].

Contamination of surface and ground waters by pathogens and chemicals tends to make domestic roof-harvested rainwater an alternative. In the study environment, most households are in the habit of harvesting and storing rainwater in jars and cisterns to supplement other sources of potable water during the raining season. Previous studies have reported on the poor microbial quality of DRHRW [13–18]. This will likely become worse if stored for a long period. However, a small increase in temperature can improve the solar disinfection effectiveness for certain microbial strains [19]. The populace is constantly faced with the problem of high cost of cooking fuels as well as diminishing wood supply. There is also the problem of overdependence on wood, leading to deforestation and its attendant problems of erosion and global warming. Other alternatives such as the use of kerosene, gas, and electricity are unattractive because they are costly, unavailable, or unreliable. Hence, this study investigated the use of locally available materials to develop solar heating system that can be used to improve the quality of domestic roof-harvested rainwater in Nigeria and other developing countries.

## 2. Material and Methods

**Figure 1:**
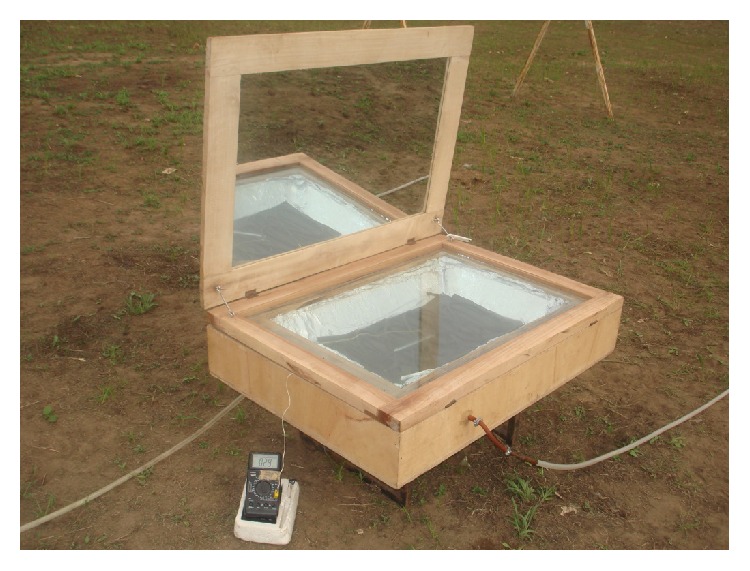
Experimental arrangement for temperature measurements (collector area, 0.47 m^2^).

The study was conducted at the University of Ibadan, Ibadan, Nigeria (latitude 7°26^1^N; longitude 3°54^1^E). Solar water heating system was designed to handle continuous flow of water with a mean design temperature of 65°C. Experimental arrangement of the solar heater for temperature measurements is shown in Figure 1. The solar water heater developed and used in this work has five important components, namely, the outer box, the inner box, double-walled glass cover, reflector lid, and absorber plate. Due to its local availability in some part of southwestern Nigeria and low thermal conductivity, coconut fiber was used as lagging material. This was introduced to fill the space between the inner and outer boxes such that there is 100 mm thickness of insulating materials all around the inner box and between the inner and outer box. The insulating material was closely packed together at about 95.58 kg m^−3^ density which is comparable to the average density of 112 kg m^−3^ recommended by Baryeh [20]. The space housing the insulating materials was then sealed up with four pieces of plywood noggins.

The solar water heating system was evaluated in an open field belonging to the Nigerian Micrometeorological Experiments (NIMEX) Research Group, Department of Physics, University of Ibadan, Nigeria, for eleven days. The meteorological instruments used comprised both slow and fast responses. These measured mean and turbulent parameters in the surface layers simultaneously. A 15 m mast was set up to measure the profiles of the mean wind speed at 0.7, 1.2, 2.2, 3.3, 5.2, 7.2, 10.2, and 14.8 m (the mean wind direction is inclusive only at the 14.8 m height) and air temperature (wet and dry bulb) at 0.9, 4.9, and 10.0 m. The same mast also supported radiation sensors for both global and net radiation at 1.5 m. The slow measurements were controlled by the use of two Campbell CR10X data loggers which sampled the data every 1 second and subsequently stored them as 1-minute averaged value. A list of all the meteorological equipment used in this study is contained in Table 1. The location was obstruction-free and free of shadows. The arrangement was to monitor the rise in temperature of the absorber plate under no-load condition. Hence, the maximum temperature attainable at a given solar radiation on a particular day can be determined. Similarly, a pyranometer was used from the same station to monitor solar radiation.

From standard procedure, thermal efficiency of a solar water heater or cooker is determined from water heating test using the relationship

(1) $$\begin{array}{c} \eta =\frac{\left(M_{w}C_{w}+M_{cu}C_{cu}\right)\sum \Delta T}{IA\Delta t}, \end{array}$$

where  *η* = thermal efficiency (%), *M*_*w*_ = mass of water (Kg), *M*_*cu*_ = mass of the copper pipe (Kg), *C*_*w*_ = specific heat capacity of water (4200 J Kg^−1^ °K^−1^), *C*_*cu*_ = specific heat capacity of copper pipe (400 J Kg^−1^ °K^−1^), *I* = total solar radiation (W m^−2^), *A* = area of absorber plate (m^2^), Δ*T* = difference in temperature (°C), and Δ*t* = difference in time (seconds).

A typical box-type heater requires adjustment every 15 to 30 min or when shadow appears on the absorber plate. The solar water heater was designed such that the orientation of the reflector lid (when operated) is facing the sunset (west). This was found to be representative of local conditions, since the users will not likely have the time to stay with the system and be turning it every 15 to 30 min. The limitation of this fixed condition is that the system will only operate effectively between the hours of 11:30 a.m. and 4:30 p.m. on clear/sunny days. However, manual azimuth adjustment will increase its efficiency. Readings were monitored and recorded at 10 min interval, similar to the method used by Mahavar et al. [21]. The highest temperature taken for the day was noted as the stagnation temperature.

Most of the studies reported in the literature on solar disinfection used water with laboratory grown organisms subjected to simulated solar irradiation [17, 22, 23]. Bacteria inactivation rate was reported to be slower for naturally occurring organisms compared with laboratory grown organisms [17]. In this study, naturally contaminated DRHRW in Ibadan, Nigeria, was harvested and released into the system at 2.61 × 10^−5^ m^3^ s^−1^ (93.96 Lh^−1^) in a continuous flow process. Water temperatures at the inlet, within the heating chamber, and at the outlet to the heating chamber and solar radiation were monitored at 10 min interval.

Samples were also collected into McCartney bottles at both the inlet to and the outlet from the heating chamber at 10 min interval for microbiological analysis. All the samples were analyzed immediately.

The total viable count was carried out by means of the standard plate count technique using plate count agar. Dilutions of water samples in buffered peptone water were inoculated by putting 1 mL into each 10 mL molten standard plate count agar in McCartney bottles. After thorough mixing, these were poured into sterile Petri dishes and incubated for 48 hours at 22°C. Petri dishes from dilutions counting 50 discrete colonies were counted and the results expressed as the number of bacteria colonies per millilitre. The isolates were further identified using their macroscopic, cultural, physiological, and biochemical characteristics. Presumptive coliform test for the detection of coliform was done after the methods stated in [24]. MacConkey broth was used for the presumptive tests. Inoculated tubes of MacConkey broth were incubated at 44°C for 2 hours. Positive presumptive tests were confirmed using eosin methylene blue agar. Colonies with characteristic growth were reinoculated in the tubes of MacConkey broth. Growth characteristics in methylene blue as well as reactions to indole, methyl red, Voges-Proskauer, and citrate utilization tests were used as confirmation of the presence of* Escherichia coli*. Mannitol salt agar was used for* Staphylococcus aureus*. The chemicals used in the study were of analytical grade and the preparation was done according to test guidelines ([24–26], API 20E and API 20NE bioMerieux, France).

## 3. Results and Discussion

### 3.1. Main Design

**Figure 2:**
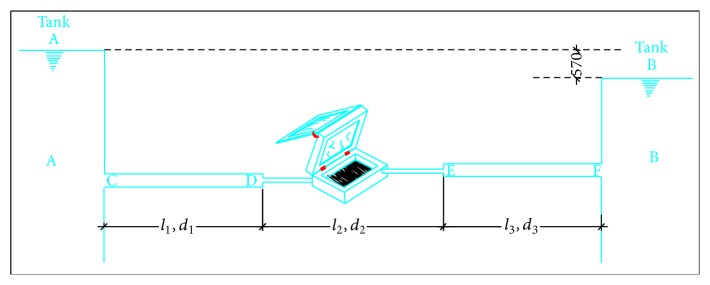
Schematic drawing of the solar heating system.

The schematic drawing for the design and fabrication of a flat plate collector, box solar water heater is presented in Figure 2. The design of the system is made such that the temperature is used up as soon as it is being built up by the incoming water that is constantly flowing through it (which is at a lower temperature, relative to the temperature within the heating chamber). The procedure for the design and fabrication of the solar water heater is presented in Figure 2.

### 3.2. Design Conditions

Design conditions are as follows:

- transparent surface area = 0.84 m × 0.56 m = 0.47 m^2^;
- absorber plate = 0.3 m × 0.6 m black coated (front and back); aluminium sheet 1.4 mm gauge was used as the absorber plate;
- absorber pipe = 4.34 m length and 0.0064 m diameter, black coated, was used as the absorber/conveyance pipe.

Aluminium plate and copper pipe were used because of their high thermal conductivity, low weight per unit area, availability, affordability, workability, and good resistance to corrosion. Aluminium is nontoxic and hence it is used in cooking ware. Corrosion of copper is most often associated with soft, acidic waters with pH below 6.5 [5]. It is only toxic at elevated concentration. It is also a micronutrient needed by the body with a dietary value of 2 mg/kg [27]. Domestic roof-harvested rainwater used in this study has mean values of pH ranging from 6.8 to 7.6, irrespective of roof types and those that were collected directly without contact with roof materials. Also, a contact time of 7 min and a temperature of less than 80°C are not sufficient to leach copper quantity that will cause toxic effect.

Aremu [28] suggested that the base area should be smaller than the surface area and that the solar water heater should be shallow enough so as to avoid side shading effect. For maximum concentration at the base of the solar water heater, a side inclination of 35° was used.

#### 3.2.1. Inner Box

Dimensions of the inner box are as follows:

- surface area = 0.84 m × 0.56 m;
- base area = 0.6 m × 0.3 m;
- sides = 0.56 m × 0.30 m × 0.17 m and 0.84 m × 0.60 m × 0.17 m (in pairs).

The inner box was lagged 0.10 m on all sides.

#### 3.2.2. Outer Box

(2) $$\begin{array}{c} \text{Dimension}\;\text{of}\;\text{the}\;\text{outer}\;\text{box}=1.07\;\text{m}\times 0.76\;\text{m}\times 0.20\;\text{m}. \end{array}$$

A reflector lid of dimension equal to the area of the lid was attached to one side of the lid, with a mirror of 0.91 m × 0.61 m, so it may act as a booster to maximize solar radiation transfer into the box.

For a flat plate collector, applying the First Law of Thermodynamics (conservation of energy),

(3) Output  energy Qa=Input  energy Qu−the  losses at  equilibriumQl.

Since the average energy from the sun is constant on a very clear day for a given locality, the energy transferred can be optimized by minimizing the heat loss (*Q*_*l*_) component.

For a double cover arrangement, Stout [29] reported that the energy input into the collector when the sun is at the zenith and its radiation is at right angle is 1 Kwm^−2^ given a cloudless sky and clear air. Approximately 12% of the energy reaching each cover is reflected for each of the glass covers in the double cover arrangement. Hence, for a solar water heater of effective surface area of 0.47 m^2^,

(4) $$\begin{array}{c} Q_{u}=1000\;\text{W}{\text{m}}^{-2}\times 0.88\times 0.88\times 0.47\;{\text{m}}^{2}=363.97\;\text{W}. \end{array}$$

If cleaned and set at the correct angle, the reflector lid (booster) can reflect about 25% of the available insolation into the system. Hence,

(5) $$\begin{array}{c} Q_{u}=363.97+\left(25\%\;\;\text{of}\;\;363.97\right)=454.96\;\text{W}. \end{array}$$

### 3.3. Heat Losses

Total heat losses from the system are the sum total of heat losses from the bottom (*Q*_*b*_), sides (*Q*_*s*_), and covers (*Q*_*c*_) given as

(6) $$\begin{array}{c} Q_{l}=Q_{b}+Q_{s}+Q_{c}, \end{array}$$

where

 
*Q*_*l*_ = heat loss  (overall, W),
 
*Q*_*b*_ = heat loss from the bottom (W),
 
*Q*_*s*_ = heat loss from the sides (W),
 
*Q*_*c*_ = heat loss from the cover (W),
  (7) $$\begin{array}{c} Q_{b}=U_{b}A\Delta T, \end{array}$$

where

(8) $$\begin{array}{c} U_{b}=\frac{K}{L}, \end{array}$$

 
*K* = thermal conductivity of lagging material = 0.0295 W m^−1^ °C^−1^,
 
*L* = thickness of lagging material = 0.10 m,
 
*A* = base area 0.60 m × 0.30 m = 0.18 m^2^,
  (9) $$\begin{array}{c} Q_{s}=U_{b}A_{s}\Delta T, \end{array}$$
 
*A*_*s*_ = area of the sides (m^2^),
  (10) $$\begin{array}{c} Q_{c}=U_{t}A_{c}\Delta TQ_{c}. \end{array}$$

From Duffie and Beckman [30],

(11) Ut=N344/TpTp−TaN+F0.31+1hw−1 N344/TpTp−TaN+F0.31+1hw−1+σ(Tp+Ta)(Tp2+Ta2) ×εp+0.0425N1−εp−1+2N+F−1εghhhεp+0.0425N1−εp−1+2N+F−1εg−N−1,

where

 
*h*_*w*_ = wind heat transfer coefficient = 5.7 + 3.8 V,
 
*N* = number of glass covers (2),
 
*ε*_*g*_ = emittance of glass (0.88),
 
*ε*_*p*_ = emittance of plate (0.95),
 
*T*_*p*_ = plate temperature.

Total heat loss was calculated to be 77.04 W.

Hence, output energy

(12) $$\begin{array}{c} Q_{a}=454.96\;\text{W}-77.04\;\text{W}=377.92\;\text{W}. \end{array}$$

The user can reckon on a total of 377.92 W, taking the losses into consideration. 1.16 W-hours is needed to heat up 1 litre of water by 1°C [28]. To heat up from ambient temperature of 30°C to 65°C, that is, temperature rise of 35°C, 1.16 Wh × 35 = 40.60 Wh. Hence, 40.60 Wh heat is required which will take 40.60 Wh/377.92 W, that is, 0.11 hr (6.45 minutes). The highest stagnation temperature recorded was 100°C.

### 3.4. Flow Rate Determination

From Figure 2, diameter (*d*_1_) = 0.0127 m = *d*_3_, *d*_2_ = 0.0064 m, length (*l*_1_) = 3.8 m = *l*_3_, and *l*_2_ = 4.34 m.

Neglecting form losses,

(13) $$\begin{array}{c} \text{discharge}\;Q_{}=Q_{1}=Q_{2}=Q_{3}. \end{array}$$

The discharge through the system was then calculated to be 93.96 Lh^−1^. To ensure the detention period of about 7 minutes within the solar panel, a serpentine or sinusoidal shape was assumed for the copper pipe used. On a clear day, the system can work effectively for about 4 hours on average, treating about 375.84 L of water. This amount should be enough to meet the minimum volume of 7.5 L per capital per day recommended by WHO [5] for a family of six, for one week.

### 3.5. Evaluation of the Solar Water Heater

**Figure 3:**
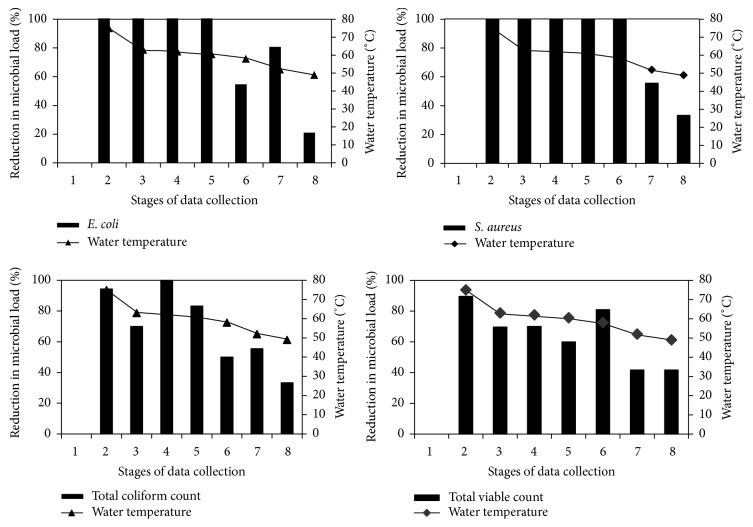
Variation in percentage reduction in microbial load with change in water temperature as a result of solar disinfection process. (1) The bar charts corresponded with the labels to the left, representing percentage reduction in microbial load. (2) The line corresponded with the labels to the right, representing water temperatures. (3) Stages of data collection refer to variation in water temperature (measured at the outlet to the heating chamber) ranging from 49 to 75°C and the corresponding percentage reduction in microbial load. Stages 2, 3, 4, 5, 6, 7, and 8 correspond with 75, 63, 62, 60.5, 58, 52, and 49°C, respectively.

The effect of temperature on microbial load of treated water with solar water heater is presented in Table 2 while the percentage reductions in microbial load due to solar disinfection are presented in Figure 3. The mean thermal efficiency of the system is 54%. The solar water heating system showed great potential for water pasteurization. It attained a maximum operating temperature of 75°C. At this temperature, a reduction of 89.6% in total viable count and 94.4% in total coliform count was achieved, while* Escherichia coli* and* S. aureus* were completely eradicated. At temperatures as low as 49°C, the heating system was still able to achieve 41.7, 33.3, 20.0, and 33.3% reduction in microbial load, respectively, for total viable count, total coliform count,* E. coli*, and* S. aureus*. Uzel et al. [31] had reported that temperature of about 60 to 70°C can prevent permanent colonization of* Legionella* spp. McGuigan et al. [32] also observed that children (5 to 16 years of age) who stored their drinking water in 1.5-litre plastic bottles that were placed in direct sunlight for continuous periods of not less than six hours in Kenya experienced a 9% reduction in incidences of severe diarrhea over three months' duration of the trial, compared with the control group. Meera and Ahammed [17], Heaselgrave et al. [22], and McGuigan et al. [23] reported the effectiveness of solar disinfection (SODIS) in disinfecting water contaminated with total coliforms: cyst of* Giardia muris* and oocysts of* Cryptosporidium parvum* poliovirus and* Acanthamoeba polyphaga*, respectively, at temperatures of 40 to 55°C.

### 3.6. Cost Implications of the Designed Solar Heating System

The averagecost of fabricating a unit of the solar water heater at the time of this research was ₦32,161.76 ($201.01) at the rate of $1 = ₦160. This amount is equivalent to the cost of purchasing 402.02 L of bottled water in Ibadan, Nigeria, where the experiment was conducted. A bottle of 1.5 L of water is being sold for about ₦120 ($0.75) in the area at the time of the experimentation. It would take the developed solar heating system 2 clear/sunny days to treat 402.02 L. Hence, the cost of constructing this system would be recovered in only 2 clear/sunny days.

## 4. Conclusion

An appropriate low cost solar heating system was developed and evaluated as a way of reducing microbiological contamination of domestic roof-harvested rainwater. The solar water heater recorded a maximum operating temperature of 75°C with 89.6 and 94.4% reduction in total viable count and total coliform count, respectively, while* E. coli* and* S. aureus* were completely eradicated at this temperature. The thermal efficiency of the solar heater was 54.0%. The DRHRW in Ibadan, Nigeria, contains some contaminants and is therefore not safe for potable uses without treatment. The solar heater developed proved to be effective in improving the quality of DRHRW. It is however not recommended for use in disinfecting water with pH less than 6.5.

## Figures and Tables

**Table 1:** List of meteorological instruments used in this study.

Parameter	Device and model	Manufacturer	Accuracy	Number
Wind speed	Cup anemometer A101ML/A100L2	Vector Instruments	Distance const. 2.3 m	10

Wind direction	Wind vane W200P	Vector Instruments	Distance const. 2.3 m	2

Air temperature (wet and dry bulb)	Frankenberger psychrometer	Theodor Friedrichs	±0.05°C	5

Surface temperature	Infrared pyrometer KT1582D	Heitronics	±0.05°C	1

Global radiation	Pyranometer SP-LITE	Kipp & Zonen	80 *µ*N/Wm^2^	1

Net radiation	Net radiometer (REBS) Q7, NR-LITE	Campbell/Kip & Zonen	+9.6 (−11.9) *μ*N/Wm^2^/13.9 *μ*N/Wm^2^	2

Source: NIMEX Research Group, Department of Physics, University of Ibadan, Nigeria.

**Table 2:** Effect of temperature on microbial load of treated water with solar water heater.

Sample	Inlet water temperature (°C)	Outlet water temperature (°C)	Microbial load cfu/mL × 10^4^							
			Total viable count		Total coliform count		*E. coli *		*S. aureus *	
			Inlet	Outlet	Inlet	Outlet	Inlet	Outlet	Inlet	Outlet
A1	32.0	75.0	48	5	18	1	10	nd	9	nd
A2	34.0	63.0	33	10	10	3	5	nd	10	nd
A3	34.0	62.0	30	9	15	nd	9	nd	7	nd
A4	32.0	60.5	48	19	18	3	10	nd	9	nd
A5	33.0	58.0	63	12	20	10	13	6	10	nd
A6	32.0	52.0	48	28	18	8	10	2	9	4
A7	32.0	49.0	48	28	18	12	10	8	9	6

nd: not detected.
